# Breast Conserving Treatment for Breast Cancer: Dosimetric Comparison of Sequential versus Simultaneous Integrated Photon Boost

**DOI:** 10.1155/2014/827475

**Published:** 2014-08-04

**Authors:** Hilde Van Parijs, Truus Reynders, Karina Heuninckx, Dirk Verellen, Guy Storme, Mark De Ridder

**Affiliations:** Department of Radiotherapy, Universitair Ziekenhuis Brussel, Vrije Universiteit Brussel (VUB), Laarbeeklaan 101, 1090 Brussels, Belgium

## Abstract

*Background*. Breast conserving surgery followed by whole breast irradiation is widely accepted as standard of care for early breast cancer. Addition of a boost dose to the initial tumor area further reduces local recurrences. We investigated the dosimetric benefits of a simultaneously integrated boost (SIB) compared to a sequential boost to hypofractionate the boost volume, while maintaining normofractionation on the breast. *Methods*. For 10 patients 4 treatment plans were deployed, 1 with a sequential photon boost, and 3 with different SIB techniques: on a conventional linear accelerator, helical TomoTherapy, and static TomoDirect. Dosimetric comparison was performed. *Results*. PTV-coverage was good in all techniques. Conformity was better with all SIB techniques compared to sequential boost (*P* = 0.0001). There was less dose spilling to the ipsilateral breast outside the PTVboost (*P* = 0.04). The dose to the organs at risk (OAR) was not influenced by SIB compared to sequential boost. Helical TomoTherapy showed a higher mean dose to the contralateral breast, but less than 5 Gy for each patient. *Conclusions*. SIB showed less dose spilling within the breast and equal dose to OAR compared to sequential boost. Both helical TomoTherapy and the conventional technique delivered acceptable dosimetry. SIB seems a safe alternative and can be implemented in clinical routine.

## 1. Introduction

For early breast cancer, breast conserving surgery followed by postoperative radiotherapy is accepted as equally effective as mastectomy [1–3]. Postoperative radiotherapy mostly consists of whole breast irradiation (WBI) with or without a boost dose to the lumpectomy area. The addition of a boost dose increases local relapse-free survival [4]. Often postoperative radiotherapy is delivered in daily fractions of 2 Gray (Gy) with a sequential boost after completion of WBI, resulting in long treatment schedules of up to 7 weeks.

In the last decades, there is a growing interest in the delivery of a simultaneously integrated boost (SIB). With this approach a daily boost dose is given to the lumpectomy area during the WBI. This results in less radiotherapy fractions. Advanced imaging techniques for accurate pretreatment staging and positioning have made it possible to more accurately delineate the boost area in the postoperative setting. With the developments in dosimetry software, an integrated boost dose within another dose volume can be calculated. Availability of daily image guidance before every radiotherapy session allows accurate repositioning of the breast and the boost volume within it.

SIB is explored earlier for other cancers [5–9], showing a good coverage of the boost region, with limited dose spread to the surrounding tissues and without excess of dose to the organs at risk (OAR). This technique is now routinely used for several indications, mainly head and neck tumors [10]. The dosimetric advantages of SIB for breast treatment also were examined earlier [11–15]. However, discussion remains on what technique is to be preferred for SIB delivery.

We present a dosimetric comparison of WBI with a sequential boost, compared to 3 different techniques of WBI with SIB.

## 2. Materials and Methods

### 2.1. Patient Selection and Image Data

From a pool of available computed tomography (CT) scans of early breast cancer patients treated in an earlier trial [16], 10 situations were selected: 5 left-sided and 5 right-sided tumors. On each side, 5 tumor locations were present: upper-outer quadrant, upper-inner quadrant, lower-outer quadrant, lower-inner quadrant, and middle of the breast. Within the selection, we included large as well as small target volumes, large as well as small breast volumes, and deeply as well as superficially located primary tumor beds on both sides. The same 10 CT scans were used for an earlier dosimetric comparison, searching for the best technique to deliver a sequential boost [17]. Patients were scanned with 3 mm slice thickness in supine position with both arms above the head in a dedicated arm support. A lead wire was placed on the surgical scar.

### 2.2. Planning Target Volumes and Organs at Risk

Contouring of the target volumes and OAR was done at the time of and according to the protocol of the phase III trial [16]. A CTVbreast was drawn to include all visible breast tissue from 5 mm under the skin to the anterior surface of the pectoralis, serratus anterior muscles. A CTVboost was drawn to include the site of the primary tumor, according to preoperative mammography and/or MRI of the breast and according to the visual seroma and/or scar on CT, with a margin of 7 mm in all directions, but within the breast tissue, to encompass potential microscopic disease extension. When present, surgical clips were to be within the CTVboost. The CTVboost excluded the skin, pectoralis muscle, ribs, lung, and heart. PTVboost to CTVboost margin was 5 mm in all directions but limited at the skin surface. The PTVbreast and PTVboost could extend beyond the pectoralis major muscle/breast tissue interface. Evaluation volumes (EV) “EVbreast” and “EVboost” were defined as the PTV limited at 5 mm below the skin surface (there was no skin involvement in these cases). The EV was used for generating dose volume histograms (DVH) and comparative analyses. A margin of 5 mm was chosen to minimize the influence of the dose build-up area at the skin, which can vary for different techniques. As OAR the ipsilateral lung, contralateral lung, heart, and contralateral breast were contoured.

- Ipsilateral and contralateral lung: autosegmentation with manual verification.
- Heart: beginning from the level in which the pulmonary trunk branches into the left and right pulmonary arteries to its most extent near the diaphragm, excluding pericardial fat.
- Contralateral breast: all visible breast tissue from 5 mm under the skin to the anterior surface of the pectoralis, serratus anterior muscles.

### 2.3. Beam Setup and Plan Prescription

In the earlier dosimetric comparison [17] we showed that the best noninvasive technique to deliver a sequential boost was with the Vero SBRT system (joint product of BrainLAB; BrainLAB AG, Feldkirchen, Germany, and MHI; Mitsubishi Heavy Industries, Tokyo, Japan) [18]. In this work only the dosimetry of the boost dose was performed. For the planning of the boost on Vero 2 conformal tangential fields (ring 0°) were chosen to cover the PTVboost and avoid as much as possible the ipsilateral lung, heart, and contralateral breast. Afterwards 2 more beams per tangential beam were added with the same gantry angle but different ring rotation (30° and 330°). As last part more conformal beams and compensation fields were added to reach a conformal dose distribution with a low dose to OAR. The maximum amount of beams per patient was kept at 10 to keep the treatment time acceptable. A treatment with 10 beams can take up to 25 minutes.

For the current comparison, we performed a summation of the dosimetry of a WBI with 2 tangential compensated fields on a conventional linear accelerator with the dosimetry of boost irradiation on Vero. This was the reference for the sequential boost technique and was compared to 3 SIB techniques, one with CMS XIO planning software (Elekta AB, Stockholm, Sweden) and 2 with the TomoTherapy system using the helical TomoTherapy as well as the static application (TomoDirect) for tangential IMRT (Accuray Inc., Madison, USA) (Table 1). For the CMS XIO planning a technique with 2 opposing tangential fields for the WBI was used. Field-in-field segments or wedges could be used to optimize dose homogeneity. For the SIB, 2 or 3 static fields or a dynamic arc technique could be used. TomoTherapy combines a rotational IMRT with a translational movement of the couch. Blocking structures and working volumes were used as was published earlier [19]. TomoDirect is the static application of TomoTherapy, where the gantry can be fixed at prechosen angles. Four tangential beams were used to cover the whole breast and 1 beam perpendicular to the breast was used to cover the boost region.

The dose prescription to the whole breast was 50 Gy, 2 Gy per fraction. For the sequential boost a dose of 16 Gy, 2 Gy per fraction, was delivered to the initial tumor bed. For the SIB a daily integrated dose of 0.4 Gy was delivered, giving a total dose of 60 Gy to the initial tumor bed. The fraction dose of 2.4 Gy was chosen to be biologically equivalent to 66 Gy in 2 Gy per fraction according to the following formula [22]:

(1) $$\begin{array}{c} \text{BED}=\frac{nd\left(1+d\right)}{\left(\alpha /\beta \right)}, \end{array}$$

where* n* is the number of fractions,* d* is the fraction dose, *α*/*β* = 3 for cosmetic outcome [23], and *α*/*β* = 4 for local recurrence [24].

The planning aims were to cover 95% of the volume of the PTV with at least 95% of the prescribed dose, but not more than 107%. For the OAR constraints and priorities were prescribed (Table 2), but an effort was made to deliver as low dose as possible. The mean near-maximum dose (D2) was used as a surrogate for the point maximum dose as suggested in the ICRU Report 83 [21].

To analyse conformity of the boost dose distribution, 2 parameters were evaluated. The mean volume of breast tissue that received 107% of the prescribed 50 Gy (Vol107) was compared as a measure of spilling of the dose by adding the boost. A conformity index (CI) was calculated, using the following formula [25]:

(2) $$\begin{array}{c} \text{CI}=\frac{{{\text{TV}}_{\text{PIV}}}^{2}}{\text{TV}\;*\;\text{PIV}\;}, \end{array}$$

where TV is the target volume, PIV is the prescription dose volume, and TV_PIV_ is the overlap of TV and PIV.

In this setting the TV is the EVboost and the PIV is the 95% isodose of the total dose (66 Gy for sequential boost, 60 Gy for SIB).

### 2.4. Statistical Analysis

The differences of means between the plans were compared and analyzed with ANOVA with a post hoc analysis test using a Bonferroni correction for multiple testing with IBM SPSS version 19.

## 3. Results

The pathological T-stage ranged from T1b to T2. The mean maximal diameter of the tumor was 1.6 cm (range: 0.6–2.7 cm). In 3 patients the deepest border of the tumor was located at a minimal distance of 3.5 cm from the skin surface. In 9 patients the PTVboost reached the skin surface. The mean PTVboost volume was 71.73 cc (range: 24.91–137.88 cc). The mean volume of the PTVbreast was 704.26 cc (range: 252.56–1234.25 cc). The mean PTVboost to PTVbreast ratio was 11.26% (range: 5.49%–19.71%). Six patients had seroma with a mean volume of 9.26 cc (range: 0.00–45.84 cc). No correlation was found between the tumor size, the PTVboost volume, or the volume of the ipsilateral breast with the CI or with the dose to the OAR.

**Figure 1:**
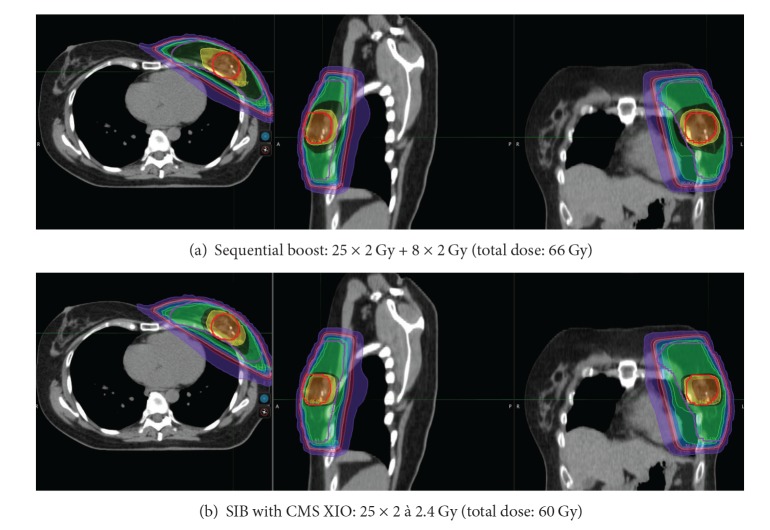
Example of dose distribution. Legend: red: 107% of total dose ((a) 70.62 Gy, (b) 64.2 Gy), orange: 100% of total dose ((a) 66 Gy, (b) 60 Gy), yellow: 95% of total dose ((a) 62.7 Gy, (b) 57 Gy), dark green: 55 Gy, middle green: 50 Gy, light green: 47.5 Gy, blue: 40 Gy, pink: 30 Gy, old pink: 20 Gy, purple: 5 Gy.

Figure 1 shows the dose distribution of the summation of the whole breast irradiation with the sequential boost on Vero compared with the SIB with CMS XIO for one patient.  Table 3 summarizes the dosimetric comparison. Coverage for all techniques was acceptable. There were a few violations (less than 95% of the EVboost or EV breast received 95% of the prescribed dose). The reason for these violations was the build-up exceeding 5 mm. In these plans there was an underdosage located at the skin.

None of the techniques achieved a CI of 70% or more. The CI was between 60 and 70% in none of the patients with the sequential technique, in 1 patient with CMS XIO, in 1 patient with helical TomoTherapy, and in 2 patients with TomoDirect. The CI was between 50 and 60% in none of the patients with the sequential technique, in 8 patients with CMS XIO, in 2 patients with helical TomoTherapy, and in 3 patients with TomoDirect. CI was less than 50% in all of the patients with the sequential technique, in 1 patient with CMS XIO, in 7 patients with helical TomoTherapy, and in 5 patients with TomoDirect. When using a cut off of 50%, there were more patients with a higher CI for the SIB techniques compared to the sequential boost. This difference was statistically significant (*P* = 0.0001). There was a statistically significant difference in the mean volume of the ipsilateral breast receiving 107% of the prescribed 50 Gy (Vol107) between the sequential boost and the SIB with CMS XIO (*P* = 0.001, Bonferroni post hoc analysis).

The difference in dose to the heart and ipsilateral lung was not statistically significant between the 4 treatment techniques. The V5 of the contralateral breast was <5% for all patients with the sequential technique, with CMS XIO and with TomoDirect. With helical TomoTherapy the V5 was >5% for 3 patients. The mean dose to the contralateral breast was low with all techniques but did show a statistically significant difference with the ANOVA test (*P* = 0.01), which could not be found with the post hoc Bonferroni testing because of the low patient numbers, though there was a trend for worse results for helical TomoTherapy (*P* = 0.069). The mean dose to the contralateral breast was less than 5 Gy for all helical TomoTherapy plans.

## 4. Discussion

The schedules and techniques for postoperative radiotherapy are developed in an era when there was no CT-scan to adjust the fields to the anatomy of the individual patient and when there were less advanced imaging techniques available to perform an accurate pretreatment staging and positioning. Electron boost dosimetry was not possible and positioning of the boost area was done clinically based upon not much more than the localization of the surgical scar and palpation of postoperative changes within the breast, guided by preoperative mammography, which deforms the breast to achieve a good image quality.

The last decades have shown a tremendous evolution in techniques for imaging and radiotherapy delivery. Yet, very often, the breast radiotherapy technique has not evolved with it. Developments in imaging techniques, dosimetry software, and treatment machines have led to the possibility of giving postoperative WBI with SIB for breast cancer patients.

The cosmetic outcome after breast conserving therapy for early breast cancer is of high importance. Less dose spilling within the treated breast theoretically diminishes the risk of developing fibrosis or late skin reactions. But a higher fraction dose could increase the risk of late skin or breast toxicity [22]. In a prospective cohort of 940 patients treated with a 3D conformal radiotherapy SIB, acceptable toxicity at a median follow-up of 30 months was shown with minimal grade 2 fibrosis in 8.5% and telangiectasia in 3.7% of patients at 3 years of follow-up [26]. In a randomized trial in our own department, we compared a long standard radiotherapy schedule of 25 fractions of 2 Gy with an additional electron boost of 8 fractions of 2 Gy to the initial tumor area with a hypofractionated schedule of 15 fractions of 2.8 Gy with a SIB of 0.6 Gy, delivering a total dose of 51 Gy to the initial tumor area. We saw equal breast symptoms [27] and equal breast fibrosis in both arms [16]. The ongoing RTOG 1005 trial will evaluate a hypofractionated dose schedule with SIB compared to a standard treatment schedule with sequential boost. The late results of these trials will deliver more information about the tolerance of SIB.

The dosimetric advantages of SIB for breast treatment were examined earlier [11–15]. We confirm these results when comparing SIB with a sequential boost, which has been performed with the best available technique according to an earlier comparison [17] and we show that advanced IMRT techniques are not required for SIB. Indeed, all SIB techniques perform better than the sequential technique. Dose distribution analysis shows more conformity and less dose spilling to the ipsilateral breast tissue outside the boost volume. This can be explained by the PTVboost being surrounded by the PTVbreast. The PTVbreast also has to receive dose, although lower than the PTVboost. In the sequential setting, dose to the surrounding breast tissue is unwanted, and in case of SIB, dose to the surrounding breast tissue is necessary. A second explanation for better conformity can be the smaller margins around the PTVboost necessary for coverage with the SIB technique than with the sequential boost. For the sequential technique, field apertures have to be several millimeters larger than the PTVboost volume to account for the penumbra. For the SIB technique no extra margin around the PTVboost is needed to obtain target coverage. This advantage was also seen by van der Laan et al. [13].

Hijal et al. compared a SIB technique with helical TomoTherapy to a 3D conformal SIB technique, showing that the helical TomoTherapy delivers better homogeneity and less dose spilling than the 3D technique [14]. For the 3D SIB technique they used 2 tangential photon fields and one perpendicular electron field.

In our comparison we find that an arc technique in most situations delivers a better homogeneity than static fields for SIB delivery. This is different in case of a sequential boost. In an earlier dosimetric comparison of different noninvasive sequential boost techniques [17], the static 3D conformal radiotherapy technique with 2 or 3 fields was preferable above arc technique. In the sequential setting, the dose spread caused by an arc technique was a disadvantage; in the SIB technique this dose spread was no longer a burden, but an advantage, provided that the dose did not spread in the direction of the lung or heart.

Franco et al. delivered SIB with TomoDirect [28]. They found mild toxicity with only 1 patient on a total of 82 patients with grade 2 or more fibrosis and good to excellent cosmesis in 75 patients at 1 year. However, a follow-up time of 1 year is not enough for cosmetic outcome. In our comparison, we find that TomoDirect is least feasible for SIB delivery. Better dosimetric results with TomoDirect are probably possible, but at the cost of longer daily treatment time and thus losing the gain in treatment time compared to helical TomoTherapy.

Compared to the earlier comparison of sequential boost techniques [17], we see lower results for CI for all SIB techniques. This is caused by the necessary presence of dose to the surrounding breast tissue. From the results of the present analysis, we feel that a CI of 50% or more should be pursued. However, there are no data available to support this. With growing experience, this may be adjusted.

For our analyses, we use the formula suggested by Paddick [25] because it considers overdosage as well as underdosage. In routine practice, however, this formula is difficult to use for decision making during the dosimetry, since it uses volumes that often can only be calculated when the dosimetry is finished. As an alternative, one could evaluate the volume within the ipsilateral breast that receives an overdosage because of the addition of the SIB, for example, the volume of the ipsilateral breast receiving 55 Gy, which is 110% or more of the prescribed dose to the whole breast. This dose is the maximum accepted dose in our department without an integrated boost. When there is a choice between several dosimetry options for SIB delivery and in case of equal dose to the OAR, the technique with the lowest V55 is to be preferred.

SIB does not cause higher dose to the surrounding OAR compared to sequential boost. We do not expect an increased cardiac or pulmonary toxicity because of a SIB, since it delivers equal dose to the heart and lungs. Thus, SIB should be equally feasible as a sequential boost for patients who benefit of a systemic treatment.

Though only low mean dose to the contralateral breast is seen, it was highest with helical TomoTherapy. This seems inherent to the rotational technique and not caused by the SIB [19]. It is however of extreme importance to apply very strict constraints in case of inverse planning. The aim should always be to deliver equal or less dose to the OAR compared to the standard technique of 2 tangential fields with electron boost. In case of multibeam IMRT, one should keep in mind that there is an increased risk for development of secondary cancers [29].

Based upon the results of this comparison, we changed the planning target values in our daily routine. We apply 2 levels of target values because we want to find the best possible dosimetry, not only a dosimetry that fits the constraints, but one that with some extra effort could be better. The first level contains very strict target values that we always strive for, derived from the mean results of the CMS XIO dosimetry in this comparison. At this level, we aim for a V30 of the heart of less than 2% for a right breast and less than 5% for a left breast irradiation. We try to keep the V10 at 0% for a right breast and less than 20% for a left breast irradiation. The mean dose should not exceed 2 Gy for a right breast and 4 Gy for a left breast. The heart has first priority, since a recent population-based case-control study showed that the rates of major coronary events increased linearly with the mean dose to the heart by 7.4% per Gy, with no apparent threshold [30].

We aim to keep the V20 of the ipsilateral lung beneath 15% when there is no lymph node irradiation and beneath 20% when the lymph node areas are part of the target. We aim for a V5 of the ipsilateral lung less than 30%. The contralateral breast is outside the field.

A minor violation exists if these very strict target values cannot be met. Minor violations are no reason to reject the dosimetry. There is a second level with absolute constraints, which are more commonly used and are based primarily upon the presumed risk of developing toxicity. The V30 of the heart should not exceed 5% for a right breast, 15% for a left breast. The maximal tolerable V10 is 10% for a right breast, 30% for a left breast, and the mean dose has to be less than 5 Gy for both sides.

The V20 of the ipsilateral lung should be less than 30%, for both lungs less than 20% with a mean dose not exceeding 15 Gy. The V5 of the ipsilateral lung should be as low as possible, but not more than 70%.

If one of these constraints cannot be met, a major violation exists. In case of one or more major violations, the treating radiation therapist has to decide if the dosimetry is clinically acceptable or should be rejected.

When a SIB is given, the lumpectomy area is irradiated with a higher dose from the start of the radiotherapy course. It is important that the delay between surgery and start of radiotherapy is long enough for postoperative changes to heal. If there is a postoperative seroma or hematoma of more than 30 cc, the boost area becomes relatively large, which has a negative impact on cosmetic outcome [31]. A delay of 3 weeks between surgery and CT-scan for dosimetry usually seems sufficient. If not, a longer delay before the start of the radiotherapy or replanning of the radiotherapy after 3 weeks is to be considered.

Next to the dosimetric advantages, SIB has practical advantages. There are less fractions, which has an economic benefit, not only for the radiotherapy department [32], but also for the patient, having less transportation costs. The daily treatment time is not majorly influenced by delivery of SIB. From our experience in the department, we estimate the routine daily treatment time on a classic linear accelerator to be 12 minutes for SIB, compared to 10 minutes for WBI without SIB. The difference in daily treatment time is mainly caused by the cone beam-CT which is performed daily in case of SIB and only during the first week of treatment for WBI without SIB. Daily treatment time for an additional boost performed with electrons takes only 5 minutes; performed with photons on Vero it can take up to 25 minutes. In case of SIB with helical TomoTherapy, there is no excess in daily treatment time. Treatment with TomoTherapy can take up to 20 minutes per session with or without SIB.

## 5. Conclusions

In conclusion, we confirm the dosimetric advantages of SIB for breast irradiation, even when compared to an advanced and highly conformal sequential technique. SIB can be performed with acceptable dosimetry on a conventional linear accelerator or on TomoTherapy. With helical TomoTherapy, there is a risk of higher dose to the contralateral breast. On a conventional linear accelerator, a technique with tangential compensated fields for WBI and arc technique for SIB is preferable in most situations. SIB seems a safe alternative and can be easily implemented in clinical routine.

## Figures and Tables

**Table 1:** Planning software and corresponding type of calculation algorithm.

Technique	Planning software	Type of calculation algorithm [20]
CMS XIO	CMS XIO Release V4.62.00.13	b
Vero	iPlan RT Dose 4.1.2 for Vero	b
Helical TomoTherapy	TomoTherapy Planning Station H-Art Version 4.0.5	b
TomoDirect	TomoTherapy Planning Station H-Art Version 4.0.5	b

**Table 2:** Constraints to organs at risk and priority list.

Priority	Organ	Constraint
1	Heart	V30 ≤ 10%
2	Ipsilateral lung	V20 ≤ 20%
3	Contralateral lung	V5 ≤ 5%
4	Contralateral breast	V10 ≤ 5%

**Table 3:** Summary of dosimetric evaluation.

	Sequential boost	SIB			Significant (ANOVA)
		CMS	Helical TomoTherapy	TomoDirect	
# violation EVboost coverage	1	2	1	1	No
# violation EVbreast coverage	2	1	0	0	No
# CI ≥ 50%	0	9	3	5	Yes (*P* = 0.0001)
Vol107 ipsilateral breast (cc)	463.32 (139.50–724.95)	230.47 (90.92–442.41)	346.61 (118.29–605.28)	365.15 (179.19–767.29)	Yes (*P* = 0.04)
D_mean_ heart left sided (Gy)	3.04 (1.30–7.35)	3.12 (1.61–7.66)	2.97 (1.59–6.89)	4.49 (1.84–8.95)	No
V5 heart left sided (%)	9.61 (3.66–23.72)	10.10 (4.29–24.70)	9.91 (2.54–25.65)	16.50 (4.82–27.27)	No
V20 heart left sided (%)	3.65 (0.83–13.20)	3.65 (0.81–13.28)	2.85 (0.00–11.10)	5.98 (0.65–15.32)	No
V30 heart left sided (%)	2.55 (0.15–10.49)	2.55 (0.04–10.60)	1.44 (0.00–6.73)	3.74 (0.32–11.79)	No
D2 heart right sided (Gy)	2.04 (1.25–3.72)	3.34 (1.17–9.52)	1.85 (1.29–2.38)	4.30 (1.11–11.52)	No
V20 ipsilateral lung (%)	10.95 (6.63–16.48)	11.15 (6.63–16.71)	10.32 (0.00–13.84)	11.33 (6.02–17.26)	No
V5 ipsilateral lung (%)	20.89 (12.59–28.87)	24.96 (12.66–47.32)	21.89 (4.36–28.49)	30.45 (16.42–61.51)	No
D_mean_ ipsilateral lung (Gy)	6.26 (3.69–8.64)	6.72 (3.73–9.20)	6.13 (2.15–7.96)	7.24 (4.85–11.14)	No
D_mean_ contralateral breast (Gy)	0.36 (0.03–1.25)	0.44 (0.14–1.45)	1.17 (0.27–3.60)	0.46 (0.18–0.56)	Yes (*P* = 0.01)
D2 contralateral breast (Gy)	2.26 (0.16–12.93)	2.39 (0.40–12.96)	4.61 (0.86–19.46)	1.19 (0.52–1.50)	No

In case more than 1 value is given, the first number is the mean value; the numbers between brackets are the minimum and maximum values.

D2: near-maximum dose, as a surrogate for the point maximum dose as suggested in the ICRU Report 83 [21].
